# Competencies for Nurses Regarding Psychosocial Care of Patients With Cancer in Africa: An Imperative for Action

**DOI:** 10.1200/GO.21.00240

**Published:** 2022-01-19

**Authors:** Marie Goretti Uwayezu, Bellancille Nikuze, Johanna E. Maree, Lori Buswell, Margaret I. Fitch

**Affiliations:** ^1^ School of Nursing and Midwifery, College of Medicine and Health Sciences, University of Rwanda, Kigali, Rwanda; ^2^ Department of Nursing Education, University of the Witwatersrand, Johannesburg & Netcare Education, Johannesburg, South Africa; ^3^ Dana-Farber Cancer Institute, Boston, MA; ^4^ Partners in Health, Boston, MA; ^5^ Bloomberg Faculty of Nursing, University of Toronto, Toronto, Ontario, Canada; ^6^ Rory Meyer's College of Nursing, New York University, New York, NY

## Abstract

Psychosocial care is considered an important component of quality cancer care. Individuals treated for cancer can experience biologic or physical, emotional, spiritual, and practical consequences (eg, financial), which have an impact on their quality of living. With the establishment of cancer centers in Africa, there is growing advocacy regarding the need for psychosocial care, given the level of unmet supportive care needs and high emotional distress reported for patients. Nurses are in an ideal position to provide psychosocial care to patients with cancer and their families but must possess relevant knowledge and skills to do so. Across Africa, nurses are challenged in gaining the necessary education for psychosocial cancer care as programs vary in the amount of psychosocial content offered. This perspective article presents competencies regarding psychosocial care for nurses caring for patients with cancer in Africa. The competencies were adapted by expert consensus from existing evidenced-based competencies for oncology nurses. They are offered as a potential basis for educational program planning and curriculum development for cancer nursing in Africa. Recommendations are offered regarding use of these competencies by nursing and cancer program leaders to enhance the quality of care for African patients with cancer and their family members. The strategies emphasize building capacity of nurses to engage in effective delivery of psychosocial care for individuals with cancer and their family members.

## INTRODUCTION

Cancer has an impact on individuals that is more than just physical; there are emotional, psychosocial, spiritual, and practical consequences as well.^1^ From the time a person thinks there may be something wrong, throughout diagnosis, treatment, and follow-up, changes occur because of the disease and its treatment, which can have a profound effect on individuals with the illness and their family members.^2^ Addressing the full range of needs patients and families experience requires psychosocial interventions in addition to those aimed at curing or controlling the disease. Evidence indicates that unmet psychosocial needs result in increased emotional distress, decreased quality of life, reduced compliance with treatments, and shortened survival.^3-7^

**CONTEXT**

**Key Objective**
This article describes competencies African nurses require to meet the psychosocial needs of patients with cancer.
**Knowledge Generated**
African patients with cancer have psychosocial needs but gaps exist in providing care to meet these needs. With relevant education, nurses are well positioned to provide basic psychosocial care and refer patients as required to psychosocial services. This article presents competencies for psychosocial nursing care for patients with cancer, which are adapted for African settings.
**Relevance**
The competencies could be used to guide development of nursing education and support nurses in providing psychosocial care to patients with cancer and family members.

Quality comprehensive cancer care, provided by an interdisciplinary team, is based on a bio-psychosocial-spiritual model and includes the provision of psychosocial care in addition to medical and surgical care.^8^ As an integral part of that team, oncology nurses in many countries address psychosocial issues for patients with cancer and their families. They are in a unique position to monitor and address patients' psychosocial distress and coping. Their education for cancer nursing prepares them for this role and enables them to contribute to achieving high-quality cancer care.^9-11^ Providing psychosocial care is embedded in the standards of practice for oncology nurses.^12-14^

With the increasing worldwide number of cancer centers being established in Africa, there is a growing advocacy to implement quality comprehensive cancer care.^15-17^ To date, much of the development of cancer care in Africa has focused on prevention of cancer and early identification and treatment of disease.^18^ Less attention has been paid to the elements of psychosocial care.^19,20^ Given the importance of psychosocial care, advocates are calling for better integration of it within daily practice.

Nurses have the potential to play important roles in providing psychosocial care across the continuum of cancer care. However, to perform that role, they require access to relevant knowledge and skills development.^21^ Caring for patients with cancer demands knowledge and skills beyond basic nursing education, clearly articulated expectations for the practice of nursing, and standards for performance on the basis of evidence.^22-24^ Relevant learning opportunities can be more easily designed for nurses when clear role expectations and competencies for patient care are established. Once nurses have the requisite education (ie, knowledge, skills, and competencies), the quality of cancer care can be enhanced.^25,26^

Although oncology nursing is a well-established specialty for nurses in the developed world, the same does not apply to Africa. Specialist nursing in Africa is most commonly on the basis of demand and supply. Irrespective of the fact that cancer is the third most common cause of mortality, oncology nursing education and training opportunities are scarce.^27^ In addition, the lack of physical resources to prevent and treat cancer, and shortages of other trained cancer care professionals, result in Africa's nurses working in conditions that will not be tolerated in the developed world.^28^

This article focuses on the role nurses can perform regarding psychosocial care of patients with cancer and their families in African settings, acknowledging other health care professionals will also be providing psychosocial care. We anticipate the description of relevant competencies could serve as a basis for curriculum design and provision of subsequent professional development programs in cancer care for nurses in Africa. Although competencies have been written about psychosocial care roles for cancer nurses in high-income countries,^12-14,29^ descriptions have not as yet been designed for the cultural and economic settings of Africa.

## METHODS

The work was undertaken by the authors who are expert oncology nurses. Three team members live and work in Africa permanently and the others have worked in various African settings as collaborators or educators as well as in other international settings. A systematic review of psychosocial needs of patients with cancer in Africa was performed and will be reported in a separate publication. Based on an understanding of gaps in psychosocial care and on personal clinical practice observations, team members identified the need for competencies related to psychosocial care to be established for nurses in Africa.

The context for this work included understanding psychosocial needs of patients with cancer across the cancer trajectory, the scope of psychosocial care for patients with cancer, the role of the nurse in cancer care, and current educational approaches for African nurses regarding psychosocial oncology. Gaps in psychosocial care and the need for development of nursing expertise in oncology nursing care were identified in various reports by psychosocial and nursing associations,^9,14,26^ and educational needs for learning oncology nursing in Africa had been reported.^24,25,27^ The context for our work is highlighted below, illustrating the need for the development of psychosocial competencies for nurses.

As an initial step, existing standards designed by oncology nursing associations were collected and reviewed by the team members independently. One member (M.I.F.) drew from the psychosocial portions of these standards documents (eg, therapeutic relationships, communication, person-centered care, and family-focused care) and designed a draft adaptation of psychosocial nursing care competencies to be used in the African context. All team members then reviewed this draft document and, through discussion, arrived at a consensus about the competencies to be included, given the African setting. It is our hope that the description of the competencies will stimulate dialogue about psychosocial care by African nurses for patients with cancer, and will serve to guide the design of new educational programs or augment content in existing programs. They may also serve to driving future research. The ultimate intention is to enhance the psychosocial care of patients with cancer and their family members in Africa.

## CONTEXT FOR THE DEVELOPMENT OF PSYCHOSOCIAL NURSING COMPETENCIES FOR AFRICAN SETTINGS

### Patients Have Psychosocial Needs Throughout the Cancer Experience

Patients with cancer experience physical, emotional, psychosocial, informational, spiritual, and practical needs throughout their cancer experience. For example, fatigue, pain, nausea, anxiety, and depression are common for patients with cancer in many countries.^30-36^ Various studies have reported needs of patients with cancer in Africa, including a range of psychosocial ones (see Table 1 for examples illustrating the range of needs identified across the psychosocial domains).

**TABLE 1:**
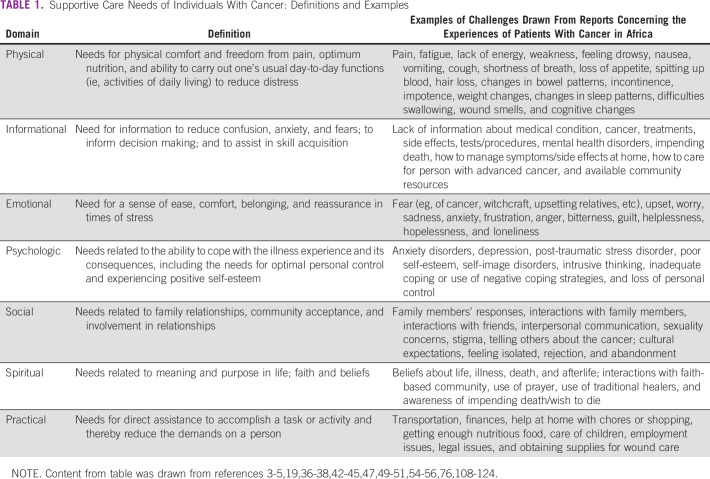
Supportive Care Needs of Individuals With Cancer: Definitions and Examples

Although patients with cancer in Africa may have similar needs to those of patients with cancer in other parts of the world,^2,30-33^ the challenges patients face in having their needs met are unique to the African setting and often exacerbated by limited resources.^36-42^ Geographical, economic, health system organization, and social and cultural factors influence how individuals are diagnosed and treated for cancer from country to country as well as supported throughout the cancer experience.^19,43^ Many African countries face significant challenges surrounding access to cancer diagnostic and treatment facilities and have high proportions of individuals diagnosed with late-stage disease.^44-46^ Issues of knowledge, transportation, personal finances, education, family support, and reliance on traditional health practices can serve as barriers to early cancer diagnosis for patients.^42,47,48^

In relation specifically to psychosocial care, investigations of emotional distress and quality of life in patients with cancer in several African countries are beginning to provide insight into patients' experience of psychosocial challenges (Table 2) and build the evidence base for practice concerning psychosocial needs. For example, Harding et al^49^ reported 75.9%% of 112 patients with cancer from South Africa and Uganda felt significant levels of sadness and 69.6% reported feeling worried. Using the same measure, Lazenby et al^50^ reported 35% of 100 patients with cancer in Botswana experienced sadness and 39% worry. Additionally, 48% of these patients experienced body image changes and 10%-29% reported high stress because of a range of bothersome symptoms.

**TABLE 2:**
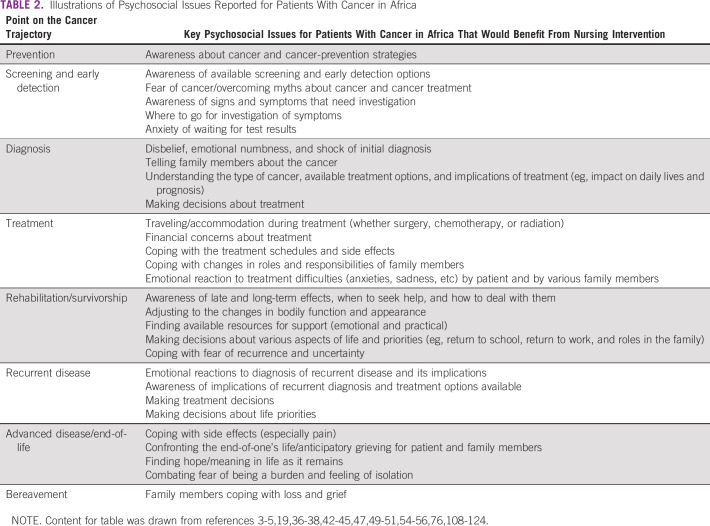
Illustrations of Psychosocial Issues Reported for Patients With Cancer in Africa

Psychosocial issues are found across the cancer continuum. For example, Clegg-Lamptey et al^3^ reported 42.7% of 89 patients with breast cancer in Ghana were frightened or terrified at diagnosis and, in a second sample of 69 women, 57.1% feared having a mastectomy.^41^ Many indicated they had had no opportunity to talk with anyone about their fear of death (61.8%) or fear of mastectomy (30.3%). Odigie et al^51^ reported that 67.9% of 81 Nigerian women felt inadequate as a woman following their mastectomy and 79.0% reported a decrease in their conjugal relations. Within three years of the surgery, 38.3% had gone through a divorce or separation from their husbands.

This same trend exists for women with cervical cancer as their challenges can start before diagnosis and last well after treatment has finished. Having cervical cancer often results in stigmatization and rejection by their communities, their families, and life partners, and the fear of treatment and death are eminent.^42,48,52^ In a study of 153 South African women with cervical cancer, Sabuleu and Maree^53^ found that all domains of quality of life were affected with social functioning being affected the most.

Clinically significant levels of psychosocial distress have also been reported for patients with cancer in Africa. For example, Fatiregun et al^54^ reported clinically significant levels of anxiety disorder in 19% of the 200 patients with breast cancer from Nigeria. Kagee et al^38^ reported significantly high levels of depression for 34.3% of 201 patients with breast cancer in South Africa, whereas Wondimagegnehu et al^55^ reported high distress for 29% of 428 patients with breast cancer in Ethiopia. Recently, Kugbey et al^56^ showed clear correlations between available social support and heightened levels of anxiety and depression for 205 patients with breast cancer in Ghana. In Morocco, Khalil et al^57^ reported in a sample of 110 survivors of cervical cancer up to 10 years post-treatment, lower emotional functioning than the control of women who had not had cervical cancer; 72% were unemployed, 31% had no interest in sexual relationships, and 41% experienced fear of recurrence.

### Psychosocial Cancer Care Defined

The domain of psychosocial care includes understanding and treating the social, psychologic, emotional, spiritual, quality of life, and functional aspects of cancer and is applied across the cancer trajectory from prevention through bereavement (Table 3).^58^ It involves attending to the needs and wishes of individuals as well as their communities of support. Psychosocial health is a state of mental, emotional, social, and spiritual well-being including the ways people view themselves and how they deal with stressful situations (Lazarus and Folkman).^59^

**TABLE 3:**
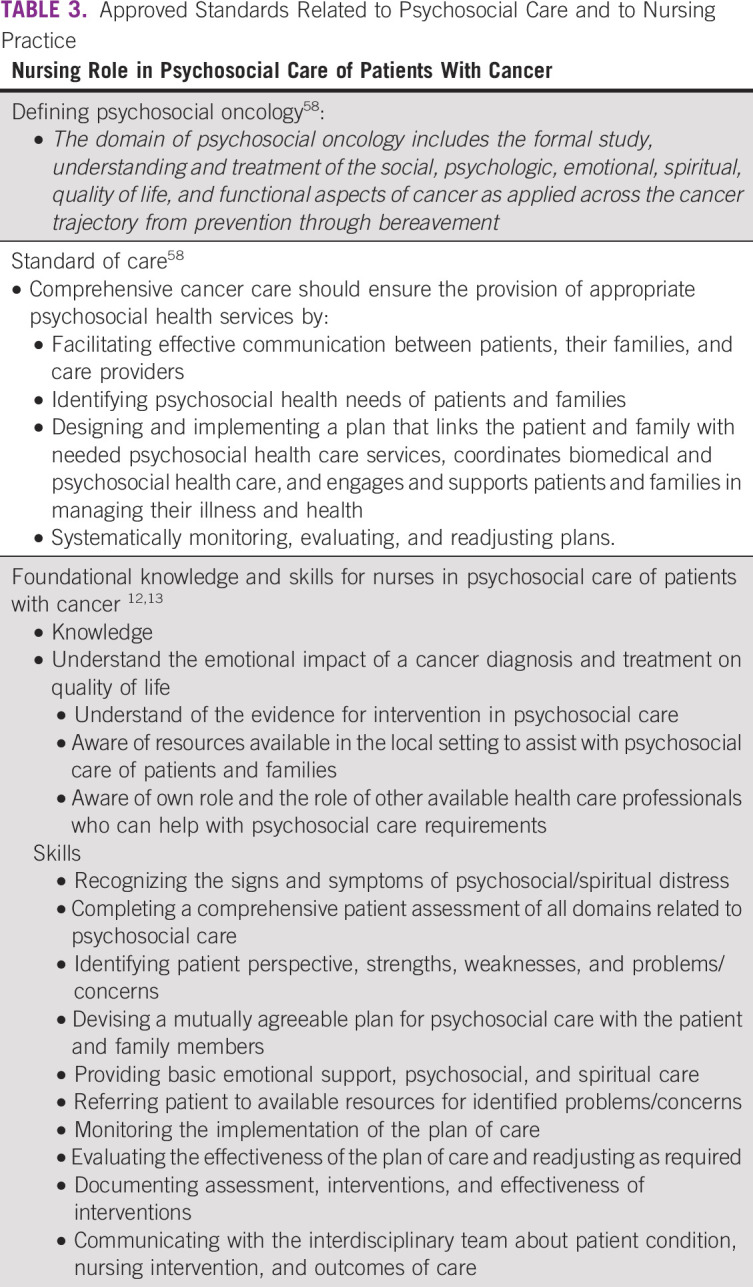
Approved Standards Related to Psychosocial Care and to Nursing Practice

Cancer and its treatment bring a myriad of changes, and coping with the consequences can be distressing for both an individual as well as family members.^1^ When human beings are confronted with stressful life circumstances, they experience emotional, cognitive, and behavioral responses unique to that individual.^59^ When cancer strikes, a person's ability to meet his or her own needs and cope may be compromised. Physical discomfort or a sense of personal crisis may interfere with an individual's usual approaches to meet his or her needs and the needs of their families. New learning, new skills, or assistance from others may be required.

All individuals will experience some degree of emotional response to their cancer diagnosis and treatment.^1^ In general, good communication, basic emotional support, and excellent symptom management will allow approximately 20% of patients with cancer to mobilize their own coping resources and manage predominately on their own.^60^ Another 30% will require additional professional encouragement and assistance concerning education about their cancer and managing side effects, access to services, and access to peer support. Meanwhile, approximately 50% will require intervention from professionals in various disciplines with expertise in cancer care to cope with the full range of unmet needs they are experiencing. Access to various health care professionals is valuable in achieving quality care outcomes for patients (ie, nutrition, social work, psychology, physiotherapy, occupational therapy, spiritual leader, and speech-language pathology). However, these services are not always available or easily accessible in many parts of Africa. Screening for psychosocial distress using a standardized tool (eg, Edmonton Symptom Assessment System-revised,^61^ Hospital Anxiety and Depression Scale,^62^ and Distress Thermometer^63^) together with a follow-up conversation can help to pinpoint the issues a patient with cancer is experiencing and mobilize appropriate psychosocial care.^64^

The principles of psychosocial care focus on caring for an individual's basic needs through adequate communication, provision of information, basic emotional support, screening of needs, and symptom management^65^ (Table 3). Higher levels of distress and unmet need will require more specialized intervention and referral to an expert in psycho-oncology.^66^ Nurses are often the preferred providers of basic psychosocial care, building on a cornerstone of therapeutic communication and response to patients' information needs and acknowledged psychosocial distress. Patients require the opportunity to talk about what is happening to them and have someone who will listen with a nonjudgmental attitude and compassion.

In summary, psychosocial care is ultimately concerned with the psychologic and emotional well-being of patients and families.^58^ It includes issues of self-esteem, insight and adaptation to illness and its consequences, social functioning and relationships, therapeutic communication, and spiritual well-being. Improvements in overall patient health require psychologic, social, and environmental interventions in addition to biomedical ones. Patients who have a good understanding of their situation and feel empowered are able to cope more effectively.

### Nursing Role in Psychosocial Care Across the Cancer Continuum

In many countries, nurses are the largest workforce in health and are often the first health care professional that individuals see when seeking help for health-related problems.^67^ The practice of nursing embraces the whole person and the person's family including all components of human need (ie, physical, social, psychologic, spiritual, and practical).^68-71^

Addressing psychosocial care needs of individuals affected by illness is a crucial role for nurses.^14,72^ If these needs are not identified and met, individuals can experience heightened psychosocial distress and poor health outcomes, while families could experience complicated grief responses.^73^ Within undergraduate preparation worldwide, nurses receive instruction, to a greater or lesser degree, on recognizing psychosocial needs and supporting patients to resolve psychosocial issues. Basic assessment of psychosocial need, problem-identification, intervention, and/or referral is expected in all nursing roles. However, providing nursing care to a specialty population, such as patients with cancer, will require additional learning and adaptation of knowledge to meet the unique psychosocial needs in the population.

Nursing care of patients with cancer is conceptualized as a specialty practice that requires knowledge and skill beyond that of undergraduate preparation.^74^ In addition to garnering additional knowledge in the disease-related interventions (ie, screening, chemotherapy administration, postoperative care, and symptom management),^23^ cancer nursing requires an astute insight into the emotional and psychosocial impact of the illness on the individual and his or her family.^75^ At present, in many parts of Africa, nurses with only undergraduate preparation are expected to perform in specialist oncology nurse roles without the benefit of additional training.^76^

Standards and accompanying competencies designed to guide cancer nursing practice have been developed in countries where cancer nursing (oncology nursing) is recognized as a specialty. Oncology nursing associations developed these standards to cover the breadth of oncology nursing care and clearly incorporate psychosocial care expectations.^12,13,77-79^ Yet, in various studies, nurses have indicated they do not feel well prepared to provide psychosocial care to patients and want added preparation.^80-82^ Describing required competencies can provide guidance regarding the added knowledge and skills to cover when preparing specialty nurses. To date, such competencies for nurses related to psychosocial cancer care have not been developed for the African context.^24^ As educational programs are established for oncology nursing in Africa, descriptions of competencies would be helpful to guide curriculum design and program implementation.

All oncology nursing standards describe expectations that nurses caring for patients with cancer will conduct basic psychosocial assessments, identify psychosocial issues or patient concerns, and provide basic emotional support. The initial expectation is directed toward creating a therapeutic relationship and providing patient support by engaging in person-centered communication. The starting point is characterized by gaining an understanding of the perspectives of the patient, through listening carefully to the individual, and acknowledging that understanding.^83^ As a basis for their interventions, nurses need to first gain an understanding of the impact the cancer diagnosis has for a patient, but then assess and monitor the ongoing impact from the patients' perspective.

An important second expectation in providing psychosocial care is identifying the concerns a patient has and providing relevant intervention and information to assist the person in coping with the situation. Having knowledge about available services that support patients in the community, connecting patients and families to necessary services, and referring individuals to relevant specialists when required are also expectations of cancer nurses. These expectations apply in any setting where nurses interact with individuals concerning cancer (eg, during public education sessions, during screening, in hospital, in ambulatory settings for chemotherapy or radiation, at home, during survivorship, and during palliative care).

Nurses prepared as specialist oncology nurses at a graduate level work with complex multifaceted and dynamic patient and family situations where conditions may be unpredictable and outcomes uncertain. The psychosocial care skills possessed by these nurses will facilitate patient and family decision making about complex treatment, symptom management, and end-of-life care. In addition, specialist nurses engage in quality improvement and evaluate service delivery, identifying gaps and relevant solutions in care. They also engage in research initiatives related to psychosocial care of patients with cancer and their families.^84^ In some settings, advanced nurses conduct support groups for patients and/or engage in counseling. They offer professional development opportunities for other nurses regarding the psychosocial care of cancer care for patients with cancer and the family members, and work with other nurses to build resources for continued learning.

In various cancer centers around the world, nurses have introduced an important role in screening for emotional and symptom-related distress using standardized distress screening tools.^85^ Tools such as the Edmonton Symptom Assessment System^61^ or the Distress Thermometer^63^ allow nurses to triage patients and use the results as a basis for a conversation about needs and supports required. Screening can help to see easily who would benefit from intervention and/or referral to other members of the cancer care team. The Distress Thermometer is beginning to be used successfully with patients who have cancer in Africa.^86^

## PSYCHOSOCIAL CARE COMPETENCIES FOR NURSES IN AFRICA

The competencies outlined in Table 4 were designed by this author team to reflect the expectations for nurses to provide psychosocial care for patients with cancer but are contextualized for the African cancer care setting. Drawn from the standards for oncology nurses, we hope the presentation of the contextualized competencies will serve to stimulate dialogue about psychosocial care by nurses and guide the development of relevant educational approaches.

**TABLE 4:**
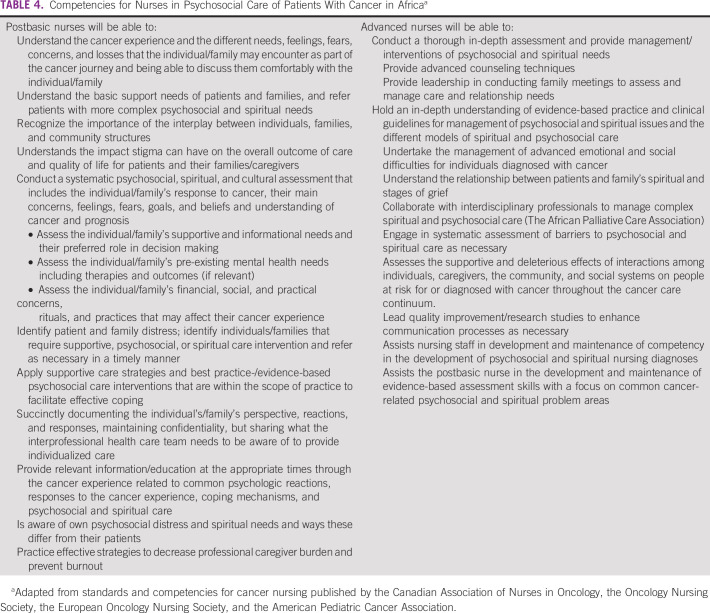
Competencies for Nurses in Psychosocial Care of Patients With Cancer in Africa^a^

The competencies cover the knowledge, skills, and values required of nurses in providing psychosocial care. Fundamental to these competencies is being able to communicate effectively in an empathetic and nonjudgmental fashion and establish a therapeutic relationship. Being able to assess psychosocial distress, identify heightened levels of distress, and know when referral is required to other disciplines are essential competencies. Providing basic emotional support, being aware of available support services for patients and families, and helping make connections to relevant services are also important.

### Nursing Education in Africa Regarding Psychosocial Cancer Care

Currently, general nursing practice standards^87-91^ in Africa align with those of the International Council of Nurses^72^ and describe expectations about providing support to patients and family members. Nurses receive basic education during their undergraduate preparation in assessing the emotional, psychologic, and spiritual needs of patients and families. However, in most countries, this education needs to be augmented^9,25,26^ for providing appropriate and meaningful psychosocial care to specialized populations such as individuals with cancer.

Currently, few formal programs exist across Africa to provide nurses with education and training in oncology nursing or specifically in psychosocial cancer care.^24,27^ Additionally, nurses in practice find it difficult to offer psychosocial care to patients with cancer because of lack of education in cancer care, unclear structures to support their practice, and lack of facility resources.^76^ Despite these challenges, there is a growing recognition across the continent that nurses can play an increasingly important role in cancer care.^92^

Presently, the role expectations for nurses working in African cancer centers vary from center to center, and very few contain explicit statements about psychosocial care by nurses.^91,93^ Few nurses have focused on psychosocial care during treatment in their research investigations which, in large measure, focus on prevention.^94^ Some cancer programs have social workers, and psychologists or psychiatrists, attached to their service^95^ or have developed support groups for patients.^96^ In some countries, there are a growing number of patient advocacy and support organizations where patients can be referred for peer-based psychosocial care and practical support.^97,98^ Additionally, the growth of palliative care and hospice have created services to assist patients in meeting psychosocial needs toward the end of life.^99,100^ Finally, patient navigation programs have been introduced recently in a few settings,^101-107^ although most focus on screening and early diagnosis.

### Proposed Recommendations to Support the Role of Nurses in Psychosocial Care of Patients With Cancer in Africa

The recommendations presented below are drawn from earlier work regarding oncology nursing as a specialty in low- and middle-income countries^9,25^ and on the basis of our understanding of the current situation in Africa and concern for psychosocial care of patients with cancer. They are offered for leaders in nursing and cancer programs across Africa to enhance the quality of care for patients with cancer and their family members. The strategies emphasize building the capacity of nurses in Africa to engage in effective provision of psychosocial care.

#### Policy

- Address oncology nursing education and training as a crucial component of health workforce development in national cancer control and implementation plans
- Provide appropriate funding for oncology nursing education programs

#### Education

- Incorporate preparation regarding competencies for psychosocial nursing care in oncology educational programs for nurses in Africa
- Develop nursing faculty who can design, implement, and teach oncology nursing both in academic and clinical settings
- Develop mechanisms for educational program sharing between countries across Africa

#### Practice

- Increase the number of specialists and clinically competent oncology nurses
- Incorporate psychosocial care explicitly into standards of practice expectations for oncology nurses
- Support consistent implementation of oncology nursing standards and competencies for practice across cancer programs, including psychosocial care

#### Research

- Develop priorities for research that will inform clinical practice, strengthen the nursing knowledge base for care of patients with cancer, advance culturally appropriate cancer care, and add to the evidence base for cancer nursing care.
- Encourage nursing research regarding the biobehavioral interventions for psychosocial and emotional needs of patients with cancer and their families in Africa.

In conclusion, this article offers a list of competencies in psychosocial care for nurses caring for patients with cancer and their families in Africa. These competencies could serve as a basis for educational program planning and curriculum development as cancer nursing grows across Africa. Both existing programs and new programs could use them to incorporate relevant psychosocial knowledge, skills, and attitudes. With preparation in psychosocial cancer care, nurses will be in a strong position to assist patients with cancer in meeting their psychosocial needs and achieving desired outcomes. Ultimately, the quality of cancer care in Africa can be enhanced.
